# Circulating Sphingolipids in Insulin Resistance, Diabetes and Associated Complications

**DOI:** 10.3390/ijms241814015

**Published:** 2023-09-13

**Authors:** Samar M. Hammad, Maria F. Lopes-Virella

**Affiliations:** 1 Department of Regenerative Medicine and Cell Biology, Medical University of South Carolina, Charleston, SC 29425, USA; 2 Division of Endocrinology, Diabetes and Medical Genetics, Department of Medicine, Medical University of South Carolina, Charleston, SC 29425, USA; 3 Ralph H. Johnson VA Medical Center, Charleston, SC 29425, USA

**Keywords:** sphingolipid, diabetes, obesity, insulin resistance, metabolic syndrome, diabetic kidney disease, cardiovascular disease, diabetic complications

## Abstract

Sphingolipids play an important role in the development of diabetes, both type 1 and type 2 diabetes, as well as in the development of both micro- and macro-vascular complications. Several reviews have been published concerning the role of sphingolipids in diabetes but most of the emphasis has been on the possible mechanisms by which sphingolipids, mainly ceramides, contribute to the development of diabetes. Research on circulating levels of the different classes of sphingolipids in serum and in lipoproteins and their importance as biomarkers to predict not only the development of diabetes but also of its complications has only recently emerged and it is still in its infancy. This review summarizes the previously published literature concerning sphingolipid-mediated mechanisms involved in the development of diabetes and its complications, focusing on how circulating plasma sphingolipid levels and the relative content carried by the different lipoproteins may impact their role as possible biomarkers both in the development of diabetes and mainly in the development of diabetic complications. Further studies in this field may open new therapeutic avenues to prevent or arrest/reduce both the development of diabetes and progression of its complications.

## 1. Introduction

### 1.1. Overview of Sphingolipid Metabolism

**Figure 1:**
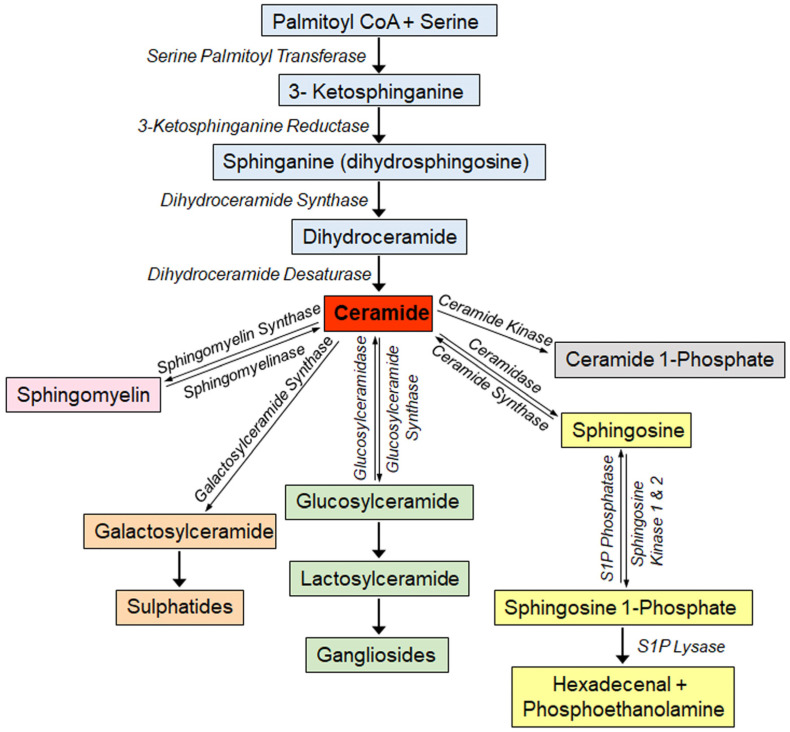
Schematic representation of the sphingolipid metabolic pathway. The de novo sphingolipid synthesis occurs in the ER, Golgi, plasma membrane, whereas the catabolic pathway (also called the salvage pathway) occurs in the acidic organelles (lysosomes and late endosomes). The enzymatic metabolism of sphingolipids occurs within a compartmentalized and interconnected network, and intersects with other metabolic pathways such as the CoA, fatty acyl, amino acid, carbohydrate, and nucleotide metabolic pathways (cellular location reviewed in, e.g., [6,7]).

Sphingolipids are structural components of cell membranes, and signaling molecules involved in the regulation of a range of cellular functions including cell growth and differentiation, proliferation, and cell death [1,2,3,4]. Sphingolipids are a diverse category of lipid molecules, which contain a backbone of sphingoid bases, which are aliphatic amino alcohols that include sphingosine or a structurally similar molecule. The sphingoid base is attached to a head group, and is N-acylated with numerous variations of fatty acids, forming diverse sphingolipid species. Ceramide, the central molecule in sphingolipid metabolism, serves as the main precursor in sphingolipid biosynthesis and can be synthesized through various pathways (Figure 1). The de novo synthesis pathway takes place in the endoplasmic reticulum (ER), where a sphingoid base can be generated by the condensation of L-serine and long-chain acyl-CoA. This is the rate-limiting step in the de novo sphingolipid synthesis and catalyzed by the enzyme serine palmitoyl transferase (SPT). The compound formed, 3-ketosphinganine, is reduced to sphinganine, to which an acyl-CoA chain will be added to form dihydroceramides, and these compounds are converted to ceramides by the enzyme dihydroceramide desaturase 1 (DES1). The addition of acyl-CoA chains to sphinganine is facilitated by a group of ER enzymes known as (dihydro)ceramide synthases (CerSs). Ceramide synthases are differentially expressed in various tissues and mediate the addition of different fatty acid chains to form dihydroceramides. The physiological function of the ceramide species is linked to the degree of saturation and the length of the fatty acid chains.

Although palmitoyl-CoA is the preferred substrate, SPT can also metabolize other acyl-CoAs, thereby forming a variety of sphingoid bases that are different in structure and function (reviewed in [5]). The mammalian (including human) SPT enzyme is composed of three subunits: SPTLC1, SPTLC2, and SPTLC3. SPTLC1 and SPTLC2 are ubiquitously expressed, but SPTLC3 expression is restricted to a few tissues. The SPTLC1 subunit is essential and can associate with either SPTLC2 or SPTLC3 to form an active enzyme. Whereas SPTLC1 and SPTLC2 mostly form C18 and C20 sphingoid bases, SPTLC1 and SPTLC3 produces a larger non-canonical variety of sphingoid bases [5]. Genetic and population studies demonstrated that SPTLC3 expression and function are associated with an altered plasma sphingolipid profile and an increased risk for cardiometabolic diseases; however, the mechanism(s) by which sphingolipids generated by SPTLC3 affects cell function is still unidentified.

Ceramide is also generated from sphingomyelin through the action of sphingomyelinases, a family of phospholipases. The ceramide formed from sphingomyelin turnover could be hydrolyzed by ceramidases to liberate sphingosine, which can be re-acylated to ceramide or phosphorylated to sphingosine 1-phosphate (S1P) by sphingosine kinase (SK) (isoforms 1 and 2) (Figure 1). Several sphingolipid metabolites (e.g., ceramide, sphingosine, S1P and ceramide 1-phosphate (C1P)) are recognized as bioactive signaling molecules [1,2,3,4,5,8,9]. In general, cellular accumulations of ceramide and sphingosine, which occur in stress responses, are associated with apoptotic responses [10]. In contrast, accumulation of S1P is usually a modulator of cell proliferation [11] and can protect cells from cell death [1].

Sphingolipids are insoluble in water, and have both hydrophobic and hydrophilic properties [12]. Alongside phospholipids, triglycerides and cholesterol, sphingolipids are transported in the blood after incorporation into lipoproteins. They are carried by low-, intermediate- and very-low-density apo B-containing lipoproteins (LDL, IDL, VLDL) and high-density lipoproteins (HDL) [6,13,14,15]. Serum albumin also binds and transports approximately one third of plasma S1P. Serum sphingolipids are less abundant than cholesterol and phospholipids, and their precise quantification is only possible using sensitive mass spectroscopy procedures. The abundant sphingolipids in the circulation are sphingomyelin, glycosphingolipids (glucosylceramide, lactosylceramide, and gangliosides) and ceramide [13]. Using HPLC-MS/MS, our group reported a comprehensive sphingolipid profile in “normal” human serum and plasma, which has since been used as a reference range for circulating sphingolipid species in healthy humans [13]. Sphingomyelin, lactosylceramide, hexosylceramide, ceramide, and C1P were found to constitute 87.7%, 5.8%, 3.4%, 2.8%, and 0.15% of total sphingolipids, respectively. The most abundant sphingolipid species are C16:0 sphingomyelin, C16:0 lactosylceramide, C24:0 hexosylceramide, and C24:0 ceramide. Effects of fasting state and sex on sphingolipid species levels were also reported [13].

Lipoprotein particles consist of hydrophobic lipids located within the core and amphipathic molecules in the surface. Complex sphingolipids exist predominantly in the hydrophobic outer layer of the lipoprotein particle with free cholesterol and phospholipids [16]. Kumpula LS et al. used a structural model to optimize the lipid distributions within lipoprotein particles based on the total molecular volumes of the core and surface [16]. They showed that the composition of the particles influences the molecular content of the surface. Using HPLC-MS/MS, our group found that the major carrier of ceramide and dihydrosphingosine in the circulation is LDL with 39.9% and 40.6% of total lipoprotein-associated ceramide and dihydrosphingosine, respectively [13]. In all isolated lipoprotein particles, C16:0 sphingomyelin was found to be the major species, followed by C24:1 sphingomyelin [13,17]. It has been also found that C24:0 ceramide is the most abundant ceramide species in all lipoprotein classes, including HDL subclasses [13,17]. The concentration of sphingomyelin and ceramide species per lipoprotein particle reflects particle size, with the larger size particle containing a higher content of sphingomyelin and ceramide species.

Studies on the flux rate of sphingolipids from tissues to plasma and the half-life of plasma sphingolipids are still limited. Recent studies on the origin of plasma sphingolipids have involved patients with abetalipoproteinemia and patients with Tangier disease [18,19]. The results identified microsomal triglyceride transfer protein (MTP) and ATP-binding cassette family A protein 1 (ABCA1) as critical determinants of plasma sphingolipid levels, and showed that MTP could be involved in ceramide and sphingomyelin secretion, but not in their synthesis. It has been also shown that ABCA1 deficiency in humans and mice reduces plasma glucosylceramide levels [19], and that ABCC10 may play a role in the synthesis and efflux of glucosylceramide to HDL particles [20].

It is established that most of the plasma S1P pool is bound to HDL particles, particularly the small HDL3 particles (ref. [13], with S1P metabolism recently reviewed in [21]). There are three main sources of S1P in blood: erythrocytes, platelets, and vascular endothelial cells. S1P is transported from erythrocytes and platelets through the Msfd2b transporter, and carried from the vascular endothelial cells through the Spns2 transporter [21]. In blood, two main acceptors transport S1P to tissues: albumin (~30% of total plasma S1P) and HDL (~60%). On HDL, S1P binds to apolipoprotein M (apo M). The preferential transport of S1P by HDL3 relative to HDL2 is due in part to the higher concentration of apo M in HDL3, and the higher affinity of S1P to HDL3 particles [21]. Noticeably, we and others did not find an association between the concentrations of total plasma S1P and HDL cholesterol [15,22]. We found, however, that total plasma S1P concentration is positively correlated with the S1P concentration in the plasma protein-containing fraction. The relative S1P content per particle was the highest in the larger VLDL particles [13], possibly explaining the positive correlations between plasma S1P with total and LDL but not HDL cholesterol reported in obesity [22].

Little attention has been paid to the extracellular transport of sphingolipids in lipoproteins, and to how that can regulate cell, tissue and organ functions. Further studies are certainly needed to determine the mechanisms by which changes in plasma sphingolipids and the sphingolipid composition of lipoproteins may contribute to alterations in the intracellular composition of sphingolipids and the development of disease.

### 1.2. Sphingolipids as Biomarkers of Disease

During the past few decades, different sphingolipids have been linked to the pathophysiology of many genetic and metabolic diseases and the role of sphingolipids in mediating disease processes is becoming evident. Detailed in vitro and in vivo investigations regarding the metabolism and functions of different sphingolipids demonstrated that different sphingolipids are involved in the regulation of cell growth and migration, inflammation, angiogenesis, apoptosis, and senescence [23]. Intracellular accumulation of sphingolipids can result from deficiencies in enzymes involved in the sphingolipid metabolism pathway (Figure 1). For example, levels of globotriaosylsphingosine (LysoGb3) were found to be increased in plasma, urine and cellular lysosomes of patients with Fabry disease [24], and can be used to confirm the diagnosis in individuals with atypical Fabry disease, and to determine the need for treatment [25]. This illustrates that measuring sphingolipid levels in the serum or other bodily fluids can predict the progression of disease and facilitate choosing the correct mode of treatment. In fact, sphingolipids have been proposed as possible biomarkers in coronary artery disease, heart failure, asthma, chronic obstructive pulmonary disease (COPD), several types of cancer, as well as Alzheimer’s disease and autoimmune diseases such as type 1 diabetes, systemic lupus erythematosus (SLE), rheumatoid arthritis and multiple sclerosis [23,26,27]. Further information regarding sphingolipids as biomarkers of metabolic diseases and how abnormal levels of different sphingolipid species found in blood, urine and cerebrospinal fluid can be important biomarkers of disease have also been extensively reviewed [23,26,28,29,30]. Relating the level of blood sphingolipids to the presence or progression of disease can possibly provide a reliable method of supporting the diagnosis and follow-up treatment, and hence improve outcomes in certain disease states. In this manuscript, we reviewed published data from our own group and from other groups on sphingolipid changes in obesity and insulin resistance, type 1 and type 2 diabetes, as well as the association of sphingolipids with the development of diabetic macro- and micro-vascular complications.

## 2. Sphingolipids, Obesity and Insulin Resistance

Obesity increases the risk for development of type 2 diabetes, non-alcoholic fatty liver (NAFL) disease, and cardiovascular diseases (CVD). Furthermore, obesity leads to the accumulation of ectopic fat in non-adipose tissues inducing insulin resistance [31]. Over the last two decades, numerous researchers linked obesity-related abnormalities in lipid metabolism and sphingolipids with the development of insulin resistance [29]. Herein, we start by reviewing the signal transduction pathways of insulin action on glucose uptake, as well as the mechanisms by which sphingolipids can interfere with the process and induce insulin resistance.

Insulin binds to the extracellular domain of the insulin receptor, inducing the activation of the intracellular kinase domains and resulting in tyrosine auto-phosphorylation of the beta subunit, which in turn initiates signaling cascades and, together with receptor binding, leads to the recruitment and phosphorylation of insulin receptor substrates [32,33,34]. IRS-1 is an important insulin receptor substrate, which upon phosphorylation, binds to the p85 subunit of phosphatidylinositol (3,4,5)-trisphosphate (PIP3) and leads to the recruitment of Akt to phosphoinositide-dependent kinase-1 (PDK1) and the mechanistic target of rapamycin complex-2 (mTORC2). This in turn leads to Akt activation, which is required to stimulate the translocation of glucose transporter type-4 (GLUT4) [35]. Insulin mediated by the activation of the PI3K-regulated pathway also influences lipid and protein synthesis as well as glycolysis and glycogenesis [36,37,38].

Ceramide accumulation has been shown to occur in skeletal muscles (cultured L6 muscle cells and C2C12 myoblasts), which were exposed to saturated fatty acids (palmitate), and cytokines (TNF). Ceramide accumulation induces protein phosphatase A2 (PP2A) activation, which in turn leads to dephosphorylation of Akt/PKB at the T308 moiety [39,40], thus reducing insulin-stimulated Akt/PKB phosphorylation. Those effects were shown to be reversed by inhibitors of ceramide synthesis such as myriocin and fumonisin B1 [41]. Accumulation of ceramide in both adipocytes and muscle cells can also block the translocation of serine/threonine kinase Akt/PKB to the plasma membrane, thus precluding its activation. Retention of PKCζ within caveolin-enriched cellular microdomains was found to be necessary to induce ceramide-mediated Akt/PKB inhibition in adipocytes and skeletal muscle [42,43,44].

Beside the direct actions of cellular ceramide accumulation on insulin resistance, recent studies strongly suggest that increased intracellular content of ceramide produced by de novo synthesis and by the hydrolysis of more complex sphingolipids (sphingomyelin, and glycosphingolipids) may also lead to insulin resistance by inducing mitochondrial dysfunction [45]. The possible mechanisms involved are still not completely understood, but several studies have shown that mitochondrial fission, increased release of reactive oxygen species (ROS), and ceramide-induced inhibition of mitochondrial respiration are involved.

In skeletal muscles of type 2 diabetes subjects [46,47,48,49], insulin-resistant offspring of type 2 diabetes subjects, and obese individuals [47], disturbance of mitochondrial respiration was observed when compared to lean normal controls. Interestingly, chronic muscle stimulation, which improves the mitochondrial respiratory function and corrects mitochondrial morphological changes [50,51], ameliorates mitochondrial function in obesity by reducing muscle ceramide content and improving insulin-stimulated glucose uptake [50].

Hammerschmidt et al. have demonstrated in a mouse model of high-fat-diet-induced insulin resistance, that ablation of ceramide synthase (CerS) 6 (CerS6) rescued the insulin-sensitive phenotype and prevented obesity [52]. The authors clearly showed that the binding of CerS6-derived C16:0 ceramide with mitochondrial fissure factor (Mff) leads to the recruitment of dynamin-like protein 1 (DRP1) to the mitochondrial surface, stimulating mitochondrial fission and leading to insulin resistance. Several other studies indicated that obesity and type 2 diabetes markedly affect redox balance [53,54,55,56]. The excess of ROS together with decreased scavenging mechanisms increase the oxidative damage of proteins, lipids, carbohydrates and nucleic acids. Therefore, excessive mitochondrial production of ROS may contribute to mitochondrial dysfunction and the inhibition of insulin action by negatively affecting insulin signal-transduction pathways [57,58].

In conclusion, ceramide accumulation directly leads to insulin resistance by affecting signal transduction pathways of insulin action and reducing glucose transport. In addition, abnormalities of ceramides and other sphingolipids induce mitochondrial dysfunction and oxidative stress, which are also well known to be implicated in obesity and insulin resistance, although the underlying mechanisms are still not well defined.

## 3. Sphingolipids and Diabetes

**Figure 2:**
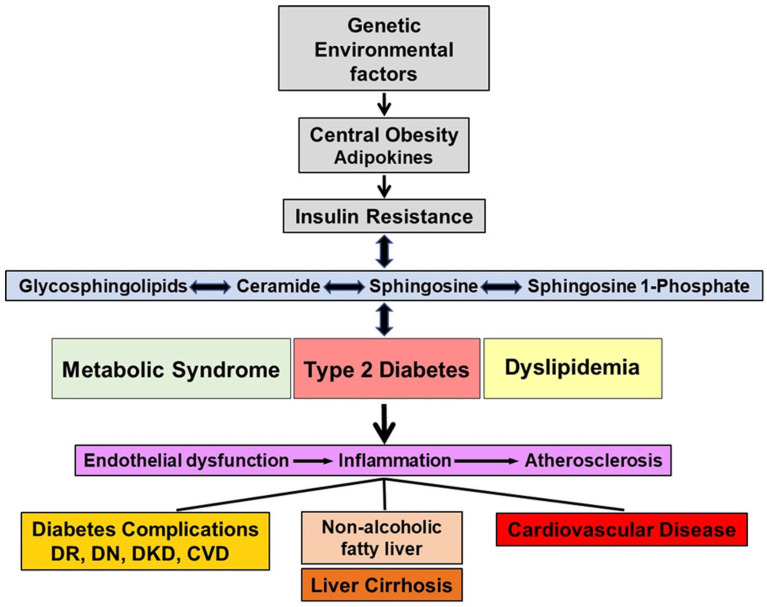
Schematic representation of the series of events mediated by sphingolipids; linking obesity to type 2 diabetes, dyslipidemia and metabolic syndrome; their contribution to endothelial dysfunction, inflammation and atherosclerosis; and the resulting complications including cardiovascular disease, diabetic kidney disease, retinopathy, neuropathy and non-alcoholic fatty liver disease/liver cirrhosis. Changes in sphingolipids can either contribute to, or result from, insulin resistance, dyslipidemia, metabolic syndrome and type 2 diabetes. DR, diabetic retinopathy; DN, diabetic neuropathy; DKD, diabetic kidney disease; CVD, cardiovascular disease.

Diabetes is a metabolic disorder that affects glucose metabolism and is characterized by hyperglycemia. There are four main types of diabetes, but in this review, we focus on type 1 and type 2 diabetes. Type 1 diabetes is an autoimmune disorder leading to beta cell destruction/damage. Type 2 diabetes is the most prevalent type of diabetes and is associated with insulin resistance and the impairment of the pancreatic beta cells. Type 2 diabetes is considered as the final stage of a metabolic disorder, which initiates with obesity and insulin resistance before progressing to prediabetes and finishing as overt diabetes. A gradual increase in glucose levels as well as lipid and sphingolipid abnormalities, which exacerbate oxidative stress and inflammation, occurs during the transition between obesity to prediabetes and during overt diabetes, both in type 1 and type 2 diabetes. In the section on type 1 diabetes, the role of sphingolipids in the autoimmune damage of pancreatic beta cells and in the development of type 1 diabetes is addressed. In the section on type 2 diabetes, we focused on reviewing the role of circulating sphingolipids in obesity, metabolic syndrome including prediabetes and type 2 diabetes. The associations of circulating sphingolipids and the development and progression of complications in both type 1 and type 2 diabetes are discussed under separate sections. Figure 2 depicts the sequence of events and the interactive relationship between sphingolipids and insulin resistance, metabolic syndrome as well as type 2 diabetes.

### 3.1. Type 1 Diabetes

Sphingolipids are known to play a pivotal role in autoimmune and inflammatory disorders [23,59]. Type 1 diabetes is an autoimmune disorder, in which auto-reactive T-cells (CD4 and CD8) produce pro-inflammatory cytokines such as TNF and IFNγ, which are able to mediate pancreatic beta cell death and activate macrophages, leading to further cytokine production and increased beta cell destruction. Recently, some investigators suggested that dietary fats and alterations in lipid metabolism including sphingolipid metabolism are involved in triggering or facilitating the autoimmune onset in type 1 diabetes, but the mechanisms involved are not yet clearly defined [60,61,62,63,64,65,66].

#### 3.1.1. In Vitro Cultured-Cell Studies

The activation of immune cells is known to trigger changes in the gene expression of proinflammatory cytokines and lead to the increased secretion of inflammatory mediators. In type 1 diabetes, most studies have focused predominantly on the release of the cytokines IL-1β, TNFα and IFNγ, and their role in beta cell damage [65,66,67,68,69,70,71,72]. Several studies clearly showed that the proinflammatory effects of cytokines on beta cells are pleiotropic, and involve disturbances of mRNA splicing, activation of several transcription factors (e.g., NFκB (nuclear factor kappa B) and AP-1 (activator protein-1 transcription factor)), changes of the expression and post-translational modifications of proteins, induction of ER stress, and mitochondrial dysfunction [61,65,68,73]. Seminal reviews on sphingolipids in inflammatory processes have been previously published [23,74,75]. Changes of sphingolipid metabolism involved in cytokine-induced beta cell death in type 1 diabetes are discussed below.

Rat insulin-producing RINm5F cells exposed for a short period of time to IL-1β were shown to have decreased sphingomyelin content and to generate ceramide and diacylglycerol (DAG), suggesting that the activation of sphingomyelinase was likely responsible for the generation of ceramide [76]. More recent studies performed in rat insulin-secreting INS1 cells, using mass spectrometry, showed that exposure to pro-inflammatory cytokines (IL-1β and IFNγ) induced neutral sphingomyelinase and activated iPLA2β, resulting in ceramide accumulation, mitochondrial decompensation and β-cell apoptosis [77]. Similar studies by Hahn et al. revealed that exposure of INS1E cells to a mixture of cytokines (IL-1β, TNFα and IFNγ) upregulated mRNA expression of acid sphingomyelinase [78]. Studies performed using a β-cell-specific iPLA_2_ β overexpressing transgenic mouse model (RIP-iPLA_2_β-Tg mice) confirmed the role of ceramide in inducing pancreatic beta cell death. This transgenic mouse model had not only a higher expression of iPLA_2_β, but also upregulation of neutral sphingomyelinase mRNA and protein expression, which resulted in a decreased sphingomyelin and increased ceramide contents [79]. Furthermore, ER stress, mitochondrial damage and caspase-3 activation were largely amplified in the transgenic mouse when compared to the wild-type control mouse [77,79].

Studies by Hahn et al. also showed that exposing pancreatic beta cells to proinflammatory cytokines upregulated various types of CerSs and, as consequence, increased cellular ceramide content and beta cell apoptosis [78]. CerS isoforms CerS2, CerS5 and CerS6, followed by isoform CerS1 are the most prevalent CerSs in rodent beta cells [28], and they generate ceramides of different chain length, with different subcellular localization (ER vs. mitochondria). Accumulation of ceramide in the ER and mitochondria may induce a stress and toxic effect contributing to beta cell dysfunction and ultimately beta cell destruction. Using the CerS6-deficient mouse model, Hammerschmidt et al. demonstrated that C16:0 ceramide generated by CerS6 could promote mitochondrial fission and insulin resistance in obesity [52]. Very-long-chain ceramides generated by the overexpression of CerS2 were shown to be associated with the activation of mitophagy and mitochondrial dysfunction [80]. It was also reported that C18:0 ceramide generated by CerS1 was able to promote LC3B-II targeting of auto-phagolysosomes to mitochondria [81], and intra-mitochondrial accumulation of C16:0 ceramide generated by CerS6 induced mitochondrial fission and mitophagy [81]. Besides inducing ER/mitochondrial stress, ceramide was shown to regulate various signaling pathways (e.g., AKT/PKB or c-Jun N-terminal kinase (JNK)) and to modulate different kinases (e.g., PKC or protein phosphatases PP1 and PP2A) (3). Moreover, elevated levels of ceramide in the plasma membrane may increase membrane rigidity, which was shown to alter signal transduction [23,60]. Several other stimuli (Fas ligand, phorbol esters, and oxidative stress) besides IL-1β and TNFα are also able to disrupt sphingolipid homeostasis [23,60].

Ceramide and sphingosine are traditionally believed to exert pro-apoptotic signals, but the actions of S1P and C1P may be either pro- or anti-apoptotic depending on the stimulatory conditions and type of tissue. Hahn et al. observed that damage of pancreatic beta cells could result from an imbalance of the enzymatic metabolic processes leading to the intracellular S1P formation (SK1 and SK2) and degradation (sphingosine phosphate lyase (SPL)) [78]. SK2, the main isoform expressed in insulin-secreting cells, is upregulated by proinflammatory cytokines [78]. In INS1E cells and rat islets, cytokines downregulate the expression of SPL, while enhancing the expression of secreted phosphoprotein 2 (SPP2) [78]. In conclusion, high ceramide, sphingosine and/or S1P concentrations may be observed in beta cells in response to cytokines. Whether intracellular S1P may or may not participate in the epigenetic regulation of genes relevant for beta cell vulnerability to the autoimmune reaction in type 1 diabetes remains to be investigated.

#### 3.1.2. Animal Studies

Confirming the role of sphingolipids in the development of type 1 diabetes are studies performed in several animal models of autoimmune diabetes such as the NOD mouse, the LEW.1AR1-iddm rat and STZ-induced autoimmune diabetic mice. The studies showed that prolonged treatment of these animals with FTY720, a high-affinity agonist of sphingosine 1-phosphate receptor 1 (S1P1) that has anti-apoptotic properties, leads to improved glycemic control and reduced immune cell infiltration and cytokine-mediated beta cell destruction [82,83,84,85].

#### 3.1.3. Human Studies

Progression of type 1 diabetes is monitored mainly by autoantibodies. Although the majority of the primary autoantibodies related to type 1 diabetes recognize peptide antigens, antibodies against lipid antigens have also been described [86]. It has been shown that around 60% of children with type 1 diabetes react against antigens composed of lysophospholipids, with many of them directed against glangliosides and sulfatides [87]. Polymorphisms in genes of the sphingolipid pathway as well as changes in serum sphingolipid profiles have also been associated with the autoimmune changes observed in patients with type 1 diabetes [86,88,89,90,91,92,93]. The ganglioside pattern has been shown to be of importance to determine beta cell susceptibility to viral infections [66,94,95,96,97,98,99,100]. Generating tools for influencing ganglioside patterns in islet cells may represent a new possible intervention to protect beta cells from cytokine and inflammation-mediated toxicity. Anti-sulfatide antibodies have been detected in newly diagnosed type 1 diabetic patients [101,102], as thoroughly reviewed by Buschard [103], and have been shown to reduce insulin secretion and exocytosis from beta cells [104]. Recently, the possibility that the cell surface sphingomyelin pattern might be involved in the autoimmune reaction against beta cells has also been proposed [105].

Orosomucoid-like (ORMDL) proteins mediate feedback inhibition of the de novo synthesis of sphingolipids by inhibiting SPT in response to elevated ceramide levels [5]. ORMDL3 is expressed in human islets and it was found to be upregulated by a mixture of IL-1β and IFNγ [73]. ORMDL3 gene polymorphism has been described in type 1 diabetes patients [88,106]. Whether this gene is expressed by beta cells and whether or not TNFα contributes to the upregulation of ORMDL3 requires further investigation.

Recent lipidomic studies of serum and blood cells from type 1 diabetes patients have shown differences in the sphingolipid profiles of type 1 diabetes subjects, when compared to non-diabetic controls [88,89,90,91,107,108]. A study by Denimal and workers investigated whether abnormalities in the HDL sphingolipid profile were present in type 1 diabetes patients with normal HDL-cholesterol concentration [109]. They did not find changes in the levels of sphingomyelin species, but found that the levels of ceramide species and S1P were decreased in HDL2 and HDL3. Some of the differences observed did not reach statistical significance [109]. The findings of Denimal et al. on ceramide species and S1P, but not those on sphingomyelin species, in HDL2 and HDL3 are in general agreement with the data reported by our group in type 2 diabetes patients with normal albuminuria [110], which is mentioned in the **Type 2 Diabetes** section below.

Most of our work on sphingolipids in type 1 diabetes has been to determine whether circulating plasma sphingolipids could predict the development of diabetic complications. This is addressed in more detail in the sections Cardiovascular Disease, Diabetic Kidney Disease, and Diabetic Neuropathy below.

### 3.2. Type 2 Diabetes

Most of the mechanisms involving sphingolipids in the development of type 2 diabetes, mainly associated with obesity, have been comprehensively reviewed in the above section on obesity and insulin resistance, and are based on data obtained in animal models, and cultured cells [6,111,112]. It is, however, important to note that, although type 2 diabetes and metabolic syndrome are usually associated with obesity or excess weight, this is not necessarily true in all cases of type 2 diabetes. Subjects with type 2 diabetes may have a normal BMI (body mass index) and in these non-obese individuals, the pathophysiology of type 2 diabetes remains poorly understood. Present consensus, still to be adequately validated, is that although the lean individuals with type 2 diabetes had a similar pathophysiology concerning their insulin resistance as the obese individual, their insulin secretory defects are much more severe. Abnormalities in plasma sphingolipid levels have long been recognized in subjects with diabetes [113] or in subjects suffering from metabolic syndrome, a set of clinical metabolic abnormalities usually associated, but not always, with obesity [114]. However, clinical consensus of what constitutes metabolic syndrome is still in flux. The pathophysiology of the several metabolic conditions incorporated under the designation of metabolic syndrome is heterogeneous and prediabetes/type 2 diabetes is considered as a metabolic continuum. This section will be mainly focused on studies, in which alterations in circulating sphingolipid levels associated with metabolic syndrome/pre-diabetes conditions and type 2 diabetes are being investigated.

### 3.3. Plasma/Serum Sphingolipidomics and Features of Metabolic Syndrome

Hanamatsu and colleagues investigated the relationship between the molecular species of sphingolipids in serum and the clinical features of metabolic syndrome: obesity, insulin resistance, NAFL disease and atherogenic dyslipidemia [115]. They found that serum concentrations of sphingomyelin species with distinct saturated acyl chains, C18:0 and C24:0, were significantly higher in the obese group than in the control group. Levels of C18:0, C20:0, C22:0 and C24:0 sphingomyelin species significantly correlated with the parameters for obesity, insulin resistance, liver function and lipid metabolism, respectively.

The biomarker project of the Midlife in the United States (MIDUS) conducted with 2063 subjects recently investigated the cross-sectional association between blood sphingolipidomic profiles and metabolic syndrome as well as other atherosclerotic risk factors [116]. This study showed that ceramide levels were positively associated with obesity, atherogenic dyslipidemia, impaired glucose metabolism, and metabolic syndrome prevalence. In contrast, hexosylceramides and lactosylceramides were inversely associated with the above biomarkers, but they were positively linked to inflammatory and vascular damage-associated biomarkers in subjects with metabolic syndrome. Recently, a comprehensive review examining sphingolipid profiling as a possible tool to stratify risk associated with the several conditions incorporated in the metabolic syndrome has been published [114].

Using hyperinsulinemic–euglycemic clamps, Tonks and colleagues compared plasma and skeletal muscle levels of sphingolipids from adiposity-matched insulin-resistant and insulin-sensitive individuals compared to a lean insulin-sensitive group [117]. Irrespective of overweight/obesity, insulin resistance in the muscle was characterized by higher levels of C18:0 sphingolipids (ceramide and ganglioside GM3); whereas in the plasma, higher levels of DAG and cholesterol esters, and lower levels of lysophosphatidylcholine and lysoalkylphosphatidylcholine indicated insulin resistance.

The impact of obesity on circulating S1P levels and its relationship with markers of glucose metabolism and insulin sensitivity were also investigated [22]. Plasma S1P levels were found to be significantly elevated in subjects with obesity compared with lean healthy subjects (~28%; *p* < 0.01), and were positively correlated with the percentage of total body fat, BMI, waist circumference, fasting insulin, insulin resistance score (HOMA-IR), HbA1c (hemoglobin A1c), and total and LDL cholesterol [22].

Lipidomic associations between prediabetes (defined as impaired fasting glucose and impaired glucose tolerance) and type 2 diabetes mellitus were examined in individuals from the Australian Diabetes, Obesity and Lifestyle Study (AusDiab) [118]. Type 2 diabetes and prediabetes were found to be positively associated with plasma levels of DAG, triglycerides and cholesterol esters as expected. Interestingly, however, both groups were also positively associated with plasma levels of ceramide and levels of its precursor dihydroceramide. Most of the significant associations in the above cohort were subsequently validated in the San Antonio Family Heart Study (SAFHS) cohort [118]. The data clearly showed that the alterations in plasma lipidome, including sphingolipidomics, usually associated with type 2 diabetes, are already present in prediabetes.

Recently, a study by Sui et al. examined serum levels of eight major sphingolipids (ceramide, glucosylceramide, lactosylceramide, sphingomyelin, sphinganine, S1P, sphingosine and sphinganine 1-phosphate) in healthy control, prediabetes and type 2 diabetes subjects at recruitment into the study and over one year follow-up [119]. Among the sphingolipids, S1P levels were associated with sex and lean mass index, but not with BMI. S1P levels were the highest in healthy controls followed by pre-diabetes and type 2 diabetes. Levels of glucosylceramide, sphingomyelin, sphinganine and sphingosine decreased in pre-diabetes compared to healthy controls and rose again in type 2 diabetes, graphically exhibiting a ‘U’ shape change during the progression of prediabetes to diabetes.

Haus and colleagues were among the first to compare the levels of ceramide species in the circulation of patients with type 2 diabetes to those in healthy controls and to determine whether they correlate with insulin sensitivity and plasma TNF concentrations [120]. Subjects with type 2 diabetes had significantly increased plasma concentrations of TNF, total ceramides, and C18:0, C20:0 and C24:1 ceramide species. The levels of the ceramide species were inversely correlated with insulin sensitivity; however, the levels of C18:1 and C18:0 ceramides were positively correlated with TNF concentrations [120]. Strong correlations between plasma ceramides and insulin resistance, particularly when considered in concert with levels of inflammatory cytokines, were also reported by other investigators [121].

The concentration of the sphingoid bases sphingosine and sphinganine (dihydrosphingosine), and several sphingolipid species were also found significantly elevated in plasma samples from type 2 diabetes patients compared to healthy control subjects [122], suggesting that the rate of cellular ceramide generation in patients with type 2 diabetes is likely elevated.

Our group has compared the plasma levels of molecular species of ceramide, sphingomyelin, lactosylceramide and hexosylceramide between a control group of normal healthy subjects with two groups of type 2 diabetes patients, one with normal albuminuria and the other with macroalbuminuria [110]. We have found that plasma levels of C18:0 ceramide were significantly higher in the two diabetes subgroups than in the control group. In contrast, C24:1 ceramide levels were significantly lower but only in the group with normal albuminuria. Plasma sphingomyelin levels were similar in all the groups studied. Among the glycosphingolipids, plasma hexosylceramide levels, like sphingomyelin levels, did not differ between controls and subjects with diabetes either with normal albuminuria or macroalbuminuria. Levels of C18:0, C20:0, C24:0 and C26:0 lactosylceramides were higher in subjects with diabetes and normoalbuminuria than in controls. Plasma levels of S1P and sphinganine 1-phosphate were lower in both groups of diabetes than in controls [110].

### 3.4. Advantage of Sphingolipidomics of Circulating Lipoproteins

Although changes in plasma sphingolipids in type 2 diabetes, mainly ceramide, have been described in several studies [6,111,121], including our own [110], the reported changes are not concordant among studies [121]. Stage of diabetes, presence or absence of complications, and measurement in total plasma/serum instead of isolated lipoproteins are likely responsible for the differences observed. Complex sphingolipids, including sphingomyelin, glycosphingolipids (lactosylceramides and hexosylceramides) and ceramides are the most abundant sphingolipids in plasma [13] and are carried predominantly by lipoprotein particles [8,12,14], whereas S1P is distributed between plasma albumin and plasma lipoproteins, mainly bound to HDL [13,15]. Therefore, if there are changes in the content of sphingolipids in the different lipoprotein particles due to alterations in the lipid/sphingolipid metabolism, these changes may not be picked up by the measurement of sphingolipids in total plasma.

Because lipoproteins (VLDL, LDL, and HDL) are the major carriers of sphingolipids in plasma, it is essential to determine sphingolipid distribution among the different lipoproteins in health and disease. The mechanisms by which sphingolipids generated at tissue level may efflux into lipoproteins or be transported to the liver for possible incorporation into lipoproteins, and the flux rate and half-life of plasma sphingolipids are mostly unknown. As previously described [19,20,21], plasma, ceramide, sphingomyelin, and hexosylceramide levels were found to be regulated by MTP and ABCA1; however, the mechanisms mediating the incorporation of lactosylceramide, dihydroceramide, as well as sphingoid bases and their phosphates into lipoproteins and their transport to the plasma are not yet identified.

Recent studies [109,123,124], including those from our own group [110], have investigated sphingolipid composition and distribution not only in plasma but also in isolated lipoproteins. We have found that levels of molecular species of ceramide and sphingomyelin in apo B-containing lipoproteins (IDL/VLDL and LDL) were not significantly different between healthy controls and subjects with diabetes and normoalbuminuria, but sphingomyelin species levels carried by LDL were increased in subjects with diabetes and macroalbuminuria when compared with the diabetes group with normoalbuminuria [110]. In contrast, the levels of C16:0 ceramide and very-long-chain ceramides carried by HDL3 and HDL2 were lower in subjects with diabetes, with either normoalbuminuria or macroalbuminuria, than in controls, with the difference being statistically significant in subjects with diabetes and normoalbuminuria [110]. This observation could indicate a diabetes-induced decrease in the tissue synthesis of ceramides leading to reduced ceramide efflux into HDL. Similar findings were observed for sphingomyelin. We found a significant decrease in all sphingomyelin species carried by HDL2 and HDL3 in the two subgroups of diabetes, either with normoalbuminuria or macroalbuminuria [110], suggesting that it is diabetes-related.

The levels of lactosylceramide species carried by LDL in both groups of subjects with diabetes did not differ from those measured in controls [110]. However, the levels of lactosylceramide species in HDL3 were decreased in both groups of diabetes compared to controls, but more significantly in the diabetes group with normoalbuminuria, mimicking the profile described above for sphingomyelin species. That could indicate less clearance/more deposition of lactosylceramides in peripheral organs. The transport mechanism of tissue sphingolipids to HDL in diabetes needs to be studied in greater detail since, except for hexosylceramide species, the levels of sphingolipids carried by HDL are consistently lower than in healthy controls. Levels of hexosylceramide species carried by LDL, either in diabetic participants with normoalbuminuria or with macroalbuminuria, did not differ from those measured in controls except for C16:0 hexosylceramide, which increased significantly (~2 fold, *p* < 0.05) in the LDL of the diabetic group with macroalbuminuria [110]. The levels of hexosylceramide species in HDL3 in diabetic participants with normoalbuminuria did not differ from those measured in controls, which is a marked difference from what was observed in the other sphingolipid classes. Only in subjects with diabetes and macroalbuminuria, the hexosylceramide levels carried by HDL3 were lower compared to controls [110]. The data suggest that diabetes does not affect hexosylceramide formation at least in the earlier or subclinical stages of kidney disease, contrary to what was observed for the other sphingolipid classes.

In the same study [110], levels of S1P carried by both HDL2 and HDL3 in the two diabetic subgroups were significantly lower and of similar magnitude. Both diabetes subgroups were under good glucose control, which may explain the similarity in S1P levels carried by HDL. S1P content in HDL has been reported to be inversely correlated with HbA1c in type 2 diabetes [125,126]. However, elevated plasma levels of S1P in type 2 diabetes have been reported in other studies [127,128]. This discrepancy may be due to the fact that the group of patients in our study had very well controlled glucose and lipid levels [110]. Both total and LDL cholesterol in the two diabetic subgroups in our study were lower than in the normal controls [110]. It is possible that the higher plasma levels of S1P previously reported were secondary to increased S1P content in apo B-containing lipoproteins not HDL.

### 3.5. Predictive Value of Plasma/Serum Sphingolipids

The circulating plasma and lipoprotein sphingolipids studies discussed above for type 2 diabetes were based on cross-sectional studies and therefore unable to provide information concerning the predictive value of sphingolipids in the development of type 2 diabetes. However, few published studies have analyzed the predictive value of plasma sphingolipid levels for the development of type 2 diabetes. In the European Prospective Investigation into Cancer and Nutrition (EPIC)-Potsdam study, the association between serum metabolites and risk of type 2 diabetes was investigated prospectively [129]. The results showed that serum levels of hexose; phenylalanine; and C32:1, C36:1, C38:3, and C40:5 diacyl-phosphatidylcholines were associated with increased risk of developing type 2 diabetes. In contrast, serum glycine; C16:1 sphingomyelin; C34:3, C40:6, C42:5, C44:4, and C44:5 acyl-alkyl-phosphatidylcholines; and C18:2 lysophosphatidylcholine were independently associated with decreased risk to develop type 2 diabetes [129]. The identified metabolites not only predicted type 2 diabetes better than traditional risk factors, but also were “further linked to insulin sensitivity and secretion in the Tübingen Family study and were partly replicated in the independent KORA (Cooperative Health Research in the Region of Augsburg) cohort” [129].

A study by Othman and colleagues in a small, homogenous population, analyzed plasma deoxysphingolipids as potential biomarkers of type 2 diabetes and metabolic syndrome [130]. They found increased concentrations of deoxysphinganine and deoxysphingosine (deoxy-sphingoid bases incorporate the amino acid alanine rather than serine) in subjects with metabolic syndrome, impaired fasting glucose and type 2 diabetes. They also found that the levels of these compounds had a significant predictive value for metabolic syndrome and type 2 diabetes [130]. In patients with diabetes, the concentration of C_16_-sphingosine, which is derived from myristoyl-CoA rather than palmitoyl-CoA, was significantly decreased. C_16_-sphingosine levels had a significant predictive value for differentiating patients with type 2 diabetes from prediabetes and metabolic syndrome [130]. Thus, it was suggested that levels of deoxysphinganine and deoxysphingosine could be a potential biomarker for metabolic syndrome and type 2 diabetes, and the levels of C_16_-sphingosine could be used to detect the progression from insulin resistance to type 2 diabetes [130].

Mounting evidence suggests that ectopic fat in liver, skeletal muscle, heart, and pancreas rather than total fat mass increases the risk for type 2 diabetes [31]. However, invasive biopsies or imaging to detect ectopic fat are clinically not practical. A cross-sectional study showed that among obese subjects, serum C22:0 ganglioside and C14:0 lactosylceramide could predict muscle triglyceride levels. Also among the obese subjects, serum C36:1 DAG and C18:4 free fatty acid (FFA) strongly predicted muscle DAG levels; whereas serum C58:5 triglyceride, C14:2 and C14:3 FFA, C38:1 phosphatidylcholine, and C24:1 cholesterol ester predicted muscle ceramide levels [131]. By contrast, among endurance-trained athletes, serum C14:1 FFA and sphingosine were significant predictors of muscle triglyceride levels [131]. The same study also showed that serum C22:0 lactosylceramide, C18:1 and C24:1 sphingomyelin together predicted insulin resistance in obese and type 2 diabetes subjects, whereas serum C50:6 triglyceride and C34:1 phosphatidylethanolamine together predicted insulin resistance in athletes [131].

In a large cohort of 3645 elderly adults (The Cardiovascular Health Study) followed from 1989 to 2015 to detect CVD, high levels of C16:0, C18:0; C20:0 and C22:0 ceramide were associated with higher risk of developing type 2 diabetes (HR of 1.21; 1.23; 1.14; and 1.18, respectively) [132]. Similar results were found in two studies involving American Indian individuals enrolled in the Strong Heart Study and in the Strong Heart Family Study [133]. In both studies, high levels of C18:0, C20:0 and C22:0 ceramide were associated with a higher risk to develop diabetes [133].

A score based on plasma concentration of C18:0 dihydroceramide, C22:1 lysoalkylphosphatidylcholine and triglyceride 16:0/18:0/18:1 was proposed and validated as a possible predictive biomarker for the development of type 2 diabetes by Mamtani and colleagues [134]. The score was a recalibrated version of the San Antonio Family Heart Study (SAFHS), and was validated in an independent cohort from the Australian Diabetes, Obesity and Lifestyle Study (AusDiab). The participants did not have type 2 diabetes at baseline and were followed-up for 23.5 years. The score predicted future development of type 2 diabetes, with 76% accuracy, independently of prediabetes. This score, when combined with risk-stratification methods currently used in clinical practice, together with metformin supplementation for high-risk individuals, was the most cost-effective strategy for type 2 diabetes prevention [134].

### 3.6. Effect of Environmental Factors

Knowledge concerning the mechanisms that regulate the levels of ceramides and other sphingolipids in response to diet and other environmental factors is limited. A study performed in subjects with obesity and type 2 diabetes investigated the effect of 12-week exercise training on insulin sensitivity and plasma ceramides and showed that the levels of plasma ceramides in subjects with obesity and normal glucose tolerance were similar to those in subjects with diabetes, in spite of differences in glucose tolerance [135]. Exercise significantly reduced body weight and adiposity and increased peripheral insulin sensitivity in both groups. Plasma C14:0, C16:0 and C24:0 ceramide levels were reduced in all subjects after the training period, and the decreases in total and C14:0 ceramide were negatively correlated with the increase in insulin sensitivity [135].

Recent work on gestational diabetes mellitus, a top risk factor for the later development of type 2 diabetes, have shown on 1035 participants of the SWIFT cohort with gestational diabetes that reduced sphingolipid metabolism, mainly related with CerS2 and CerS4 genes, was highly associated with the development of type 2 diabetes in patients with gestational diabetes [136]. Also, recently, it has been reported that sphingomyelin profiling in patients with newly diagnosed diabetes could be potentially helpful to perform differential diagnosis of type 1 diabetes, latent autoimmune diabetes in adults (LADA), a slow-progressing form of autoimmune diabetes and of challenging cases of type 2 diabetes [137].

In conclusion, ceramides, sphingomyelin and glycosphingolipids have been associated with obesity, metabolic syndrome, prediabetes and type 2 diabetes; and some of the sphingolipid species seem to be valid predictors for the development and/or progression of diabetes and of its complications. Considering the complexity of the auto-immune process in type 1 diabetes, and the heterogeneity of the pathophysiology of metabolic syndrome and type 2 diabetes, more studies using homogeneous and well-characterized populations are certainly needed. Moreover, sophisticated methodology, such as measurements of the sphingolipid content of the different lipoproteins, are needed to reach a consensus concerning the association and/or predictive value of sphingolipids in the development and progression of type 2 diabetes and the metabolic syndrome. A comprehensive sphingolipid profile is certainly more informative about metabolic syndrome than ceramides alone and provides further insights into the pathophysiology of diabetic versus cardiovascular risk in patients with metabolic syndrome. Further investigations concerning the mechanisms mediating the effects of extracellular sphingolipids on cells, tissues, and organs are certainly indispensable. Since current cholesterol-regulating therapeutics, including statins have limited and indirect effects on sphingolipid metabolism and transport, further investigations may lead to novel sphingolipid-mediated intervention, which may lessen the remaining residual risk for the development of vascular complications in diabetes.

## 4. Cardiovascular Disease

Alterations in the distribution and concentration of plasma sphingolipids have been shown to be compellingly associated with the pathogenesis of atherosclerosis and CVD [138]. Abnormalities in sphingomyelin, ceramide and glycosphingolipids have been associated with increased atherosclerosis.

### 4.1. Human Studies

#### 4.1.1. Cardiovascular Disease without Diabetes

Higher plasma levels of sphingomyelin have been proposed as independent risk factors for coronary heart disease in human subjects [139]. In atherosclerotic plaques, LDL has been found to have a higher content of sphingomyelin compared to plasma LDL, mainly arising from de novo synthesis in the aorta [140]. Sphingomyelinases may hydrolyze LDL-sphingomyelin in the arterial wall increasing LDL-ceramide and resulting in aggregation of lipoproteins, which like LDL-containing immune complexes, leads to the initiation and progression of atherosclerosis [141].

Using unbiased machine learning to identify sphingolipid species positively associated with coronary artery disease, certain ceramide species together with other less abundant lipid molecules were found to be predictive of cardiovascular death in coronary artery disease patients independently and more effectively than conventional clinical CVD biomarkers including serum LDL cholesterol [142,143]. The ceramide risk score CERT1 (cardiac event risk test 1) that was developed by Zora Biosciences [144] is in operation at the Mayo Clinic as a means to predict 5-year risk of cardiovascular mortality [142,145].

In the Cardiovascular Health Study (CHS) cohort, plasma ceramides and sphingomyelins with very-long-chain of saturated fatty acids were found to be associated with reduced risk of incident atrial fibrillation [146] and incident heart failure [147]. However, ceramides and sphingomyelins with C16:0 are associated with higher risks of atrial fibrillation and heart failure. As mentioned above, ceramide plays a role in apoptosis [138,148], which is involved in heart remodeling and fibrosis leading to both heart failure and atrial fibrillation [149,150]. It has been recently reported that reduced risk of total mortality is associated with increased levels of plasma C22:0 and C24:0 ceramides, and increased levels of sphingomyelins with very-long-chain of saturated fatty acids; however, increased mortality is associated with increased levels of ceramide and sphingomyelin with C16:0 [151].

A comparative metabolomic and lipidomic study in obese middle-aged men and normal weight controls conducted over a period of three years showed that increased plasma levels of lactosylceramide, L-octanoylcarnitine, systemic blood pressure, and BMI were independent predictors of arterial stiffness/cardiac dysfunction [152]. Additionally, an age-related increase in plasma L-octanoylcarnitine, lactosylceramide, systolic blood pressure, and baseline BMI were shown to be independent predictors of increased arterial stiffness [152]. Apostolopoulou et al. also reported that, in insulin-resistant obese subjects, the serum and liver levels of lactosylceramides were markedly increased in the subjects with NAFL disease, when compared with those without NAFL, those without non-alcoholic steatohepatitis (NASH), or the healthy lean controls. Interestingly, the liver levels of total lactosylceramides and the C24:1 lactosylceramide species were not significantly higher in subjects with NASH than in the healthy lean controls after adjustment for BMI [153]. This study failed to mention which species of serum lactosylceramides were increased. The correlations of several species of lactosylceramides with cardiometabolic disease are different. For instance, high levels of C16:0 lactosylceramide are associated with pro-atherogenic effects, insulin resistance and the development of NASH. In contrast, high levels of long-chain lactosylceramides (C24-C26) are associated with anti-atherogenic outcomes. This point is discussed in more detail below under animal studies.

#### 4.1.2. Cardiovascular Disease with Diabetes

Our data concerning plasma glycosphingolipids in type 1 diabetes (unpublished data) and that obtained in SLE patients, another autoimmune disease with CVD complications, clearly show a decrease rather than an increase in plasma lactosylceramide levels. In the SLE study, the area of atherosclerotic plaques was inversely correlated with lactosylceramide concentrations at study baseline [154]. Remarkably, there was no correlation between concentrations of LDL-cholesterol, the traditional risk factor for CVD, and lactosylceramide concentrations [154], further suggesting that sphingolipids are independent risk factors of CVD and may be part of the CVD residual risk that remains after statin therapy, as proposed by Chapman [124].

Interestingly, our data obtained in the DCCT/EDIC cohort also showed that low levels of lactosylceramides are associated with the development and progression of CVD, defined by serial measurements of carotid intima thickness performed during a 25-year follow-up period. Low levels of several species of lactosylceramides were predictive of carotid intima-media thickening progression, independently of other risk factors, such as hypertension, lipid levels, and HbA1c (Lopes-Virella unpublished data). This is not surprising considering the close relationship between CVD and diabetic kidney disease and the fact that low levels of long- and very-long-chain lactosylceramides were also predictive of development and progression to diabetic kidney disease in the same cohort as recently published [155].

In an earlier study, Hammad et al. showed that healthy African Americans have lower plasma lactosylceramide levels compared to healthy whites [156], and the more recent data [154] showed that plasma lactosylceramide levels in the African Americans with SLE correlate negatively with the area of atherosclerotic plaques. This observation raised the question whether African Americans have an inherent tendency towards increased accumulation of lactosylceramides in tissues or having reduced efflux of lactosylceramides from tissues into circulating lipoproteins.

The data on auto-immune disorders (type 1 diabetes and SLE) suggest that the association of low circulating levels of some lactosylceramide species with the development of complications differ from similar associations described in other metabolic disorders. Our studies in type 1 diabetes show that low lactosylceramide levels, at a very early stage of type 1 diabetes, were a strong predictor for the development and progression of both CVD and diabetic kidney disease. Further studies are needed to better understand the role of circulating sphingolipids in predicting disease development and progression. It is noteworthy to remember that total plasma levels of sphingolipids do not necessarily translate differences in the content of sphingolipids in individual plasma lipoproteins. The content of sphingolipid species carried by the different lipoproteins can more reliably be associated with the development of CVD and other complications of diabetes [110]. Importantly, race, age, stage of disease, and treatment history are crucial factors to be considered and they should always be an integral part of any study design involving plasma/lipoprotein sphingolipidomics as diagnostic, prognostic, and targets for therapy.

### 4.2. In Vitro Cultured-Cell and Animal Studies

Experimental myocardial infarction in male Wistar rats showed significant alterations in the levels of sphingomyelin in plasma, erythrocytes and platelets [157]. Increased plasma sphingomyelin levels have been also reported in apo E knockout mice compared to wild-type mice [158]. Furthermore, in mice, overexpression of sphingomyelinase 2 (SMS2), the enzyme that generates ceramide, was found to exacerbate the inflammatory process in atherosclerosis [159], whereas inhibition of sphingolipid synthesis by myriocin reduced atherosclerosis [160].

Like sphingomyelin, increased plasma and aortic ceramide levels are associated with increased risk of CVD [160], possibly by promoting lipoprotein aggregation and enhanced inflammation and apoptosis, therefore leading to plaque instability. In vitro, ischemia/reperfusion of rat hearts were associated with decreased levels of sphingomyelin and significantly increased ceramide concentrations [161]. It has been shown that ceramide-induced apoptosis of cardiomyocytes may result from TNF-α-induced synthesis of ceramide (reviewed in [162]).

In contrast to ceramide and sphingomyelin, plasma S1P is believed to be cardio-protective (reviewed in [138]). In patients, plasma levels of S1P significantly decrease after myocardial infarction [163] and increase after percutaneous coronary intervention [164]. Sattler et al. showed in vitro that low S1P levels are associated with impaired cell signaling and vasodilation, but these defects can be corrected by loading HDL with S1P [165], indicating that low S1P could be a contributing factor of HDL dysfunction in atherosclerosis. Data from several studies on CVD in diabetes support the idea that S1P and ceramide have reciprocal actions in muscle, cardiomyocytes and the vascular endothelium (reviewed in [166]). However, studies in the liver showed that ceramide and S1P do not always exhibit reciprocal actions. Several studies suggest that S1P may contribute to the metabolic pathologies associated with obesity (reviewed in [167]). Levels of S1P were found to be elevated in obesity [22], and a substantial body of literature suggests that elevated S1P levels mobilize immune cells and enhance liver fibrosis, thus contributing to NAFL disease [167,168,169,170,171,172]. Additional studies are needed to determine the exact role of S1P in diabetes and in its macro- and micro-vascular complications.

Adiponectin, by stimulating its receptor’s inherent ceramidase activity, leads to the formation of sphingosine, which is then phosphorylated via SK to produce S1P [173]. Since adiponectin levels are low in diabetes [174], this leads to an increase in tissue ceramides and a decrease in S1P levels [162]. There is an overlap between inflammation and ceramide production converging on the TLR4 pathway [175]. A subset of fatty acids (mostly saturated) that activate TLRs induce ceramide synthesis [41,176]. The TLR4-mediated pathway was found to mediate ceramide production via the activation of sphingomyelinase [177].

Glycosphingolipids were shown to accumulate in atherosclerotic lesions both in humans and apo E knockout mice [178,179]. In animal models of diabetes, administration of glycosphingolipid inhibitors improved glucose intolerance [180,181,182], suggesting a causative role. In a mouse model of atherosclerosis, apo E knockout mice fed a high-fat and high-cholesterol diet (Western diet) had increased blood levels of lactosylceramide, and were correlated with increased arterial stiffness and aortic intima-media thickening [182]. Inhibiting glycosphingolipid synthesis not only lowered the level of lactosylceramide and glucosylceramide but also improved arterial stiffness and aortic intima-media thickening [182]. Using a biopolymer-encapsulated D-PDMP (D-thero-1-phenyl-2-decanoylamino-3-morpholino-1-propanol, a glycosphingolipid synthesis inhibitor), a recent study in type 2 diabetes (*db/db*) mice showed that reducing lactosylceramide levels may be sufficient to reduce blood glucose, cholesterol, and triglyceride-rich lipoproteins and reduce body weight [183]. The above studies postulated that increased levels of lactosylceramides may lead to superoxide generation and activation of multiple signaling pathways leading to inflammation, proliferation, adhesion, migration, angiogenesis apoptosis and increased oxidative stress [183,184]. These studies, however, did not consider which lactosylceramide species were increased. A study conducted recently clearly shows that increased levels of different species of ceramides lead to opposite effects in the development of steatosis and NAFLD [185]. This study showed that mice placed on a high-fat diet to develop steatosis and NAFLD had significantly decreased levels of CerS2, which generates C22:0-24:0 ceramides, and increased levels of CerS6, which generates C16:0 ceramide. Experiments performed in Hep3B cells overexpressing or lacking these synthases clearly showed that overexpression of CerS 6 and knock down of CerS2 led to increased hepatic ER stress and increased lipogenesis [185]. Therefore, which sphingolipid species is/are altered needs to be determined to assess their pathogenic potential.

## 5. Diabetic Kidney Disease

Diabetic kidney disease (diabetic nephropathy) is the major cause of end-stage renal failure and the main contributing factor, together with CVD, of increased morbidity and mortality in both type 1 and type 2 diabetes [186,187]. Abnormalities in sphingolipid metabolism are well-known contributing factors to the development of diabetes and its complications [111,155,188,189,190]. Diabetic kidney disease is the leading cause of chronic kidney disease, and it is closely associated with increased risk for CVD [191]. Until recently, most of the published studies were performed in animal models and it was postulated that hexosylceramides and lactosylceramides are synthesized within the kidney and accumulate in the kidney leading to organ damage. Recent studies conducted in humans strongly suggest, however, that circulating sphingolipids/glycosphingolipids also reflect or predict kidney damage [110,113,155,189].

### 5.1. In Vitro Cultured-Cell and Animal Studies

Glycosphingolipids are abundant in kidney podocytes, mesangial cells, and tubular epithelial cells, and are involved in kidney metabolism and functionality [192]. An early study in streptozotocin-treated diabetic mice, a well-known model for type 1 diabetes, showed increased glycosphingolipid deposition in association with renal hypertrophy [193]. The authors postulated that “kidney deposition of glycosphingolipids was responsible for the renal hypertrophy”. They administered PDMP [193], a ceramide analog that inhibits lactosylceramide synthesis, to the streptozotocin-treated diabetic mice and were able to show reversal of glomerular hypertrophy. They also postulated that the “formation and deposition of glycosphingolipids may be dependent on the degree of hyperglycemia”, thus creating a novel link between glucose levels and the development of nephropathy in diabetes [193].

To investigate the mechanisms by which glucose leads to the increased accumulation of glycosphingolipids in the kidney, Subathra et al. [194] exposed mesangial cells to glucose levels, similar to those found in patients with diabetes, and demonstrated that the cells became hypertrophic, with increased levels of hexosylceramides and deposition of extracellular matrix proteins. They also reported that, in the presence of a glucosylceramide inhibitor or lowering of glucose levels, mesangial cell hypertrophy was reversed and that was secondary to the decreased activation of the signaling pathways Smad3 and Akt [194].

Although glucose is needed for the formation of glycosphingolipids and elevated glucose levels enhance the accumulation of glycosphingolipids in the kidney, elevated glucose is not the only possible mediator leading to increased glycosphingolipids accumulation in kidney disease. Studies performed in animal models of lupus nephritis showed a similar pattern of kidney disease with increased accumulation of glycosylceramides and lactosylceramides [195].

Generation of ROS could be a possible mechanism contributing to glycosphingolipid deposition in the kidney. In diabetes, as well as in other inflammatory diseases, the generation of ROS is markedly increased [196,197,198,199]. ROS were found to regulate neutral sphingomyelinase 2 (nSMase2) activity, which may well affect glycosylceramides synthesis/breakdown [196,200]. ROS may also affect the activities of ceramidase and sphingomyelin synthase, and therefore the formation of glycosphingolipids found associated with aging-related inflammation [201]. Enhanced breakdown of complex glycosphingolipids cannot be excluded as another possible mechanism contributing to accumulations in hexosylceramides/lactosylceramides in the kidney. Therefore, alterations in sphingolipids by several possible different mechanisms or a combination of mechanisms may contribute to complications of diabetes, specifically to the development of diabetic kidney disease.

### 5.2. Type 1 Diabetes Human Studies

In type 1 diabetes, several cross-sectional studies were performed. A cross-sectional study was performed as part of the Finnish Diabetic Nephropathy Study on a subgroup of 325 patients with type 1 diabetes [202]. In this study, higher sphingomyelin levels were found to be associated with kidney disease, and sphingomyelin was the strongest biochemical covariate of albumin excretion rate (AER), followed by very-large and large VLDL particles [202]. Recently, in a prospective setting, the same group showed that serum sphingomyelin levels are positively associated with a rapid decline of eGFR (estimated glomerular filtration rate) and progression to ESRD (end-stage renal disease) in type 1 diabetes, and concluded that high sphingomyelin levels, independently of classical lipid risk factors, may contribute to the initiation and progression of kidney disease [203]. In contrast, in another cross-sectional study on a cohort of type 1 diabetes subjects followed at the Steno Diabetes Center, Copenhagen, between 2009–2011, a strong negative association of serum sphingomyelin levels with eGFR and macroalbuminuria was reported [204]. In longitudinal analyses, higher serum levels of sphingomyelin and phosphatidylcholine species, independently of traditional markers of kidney function, were found to be associated with a lower risk of a combined renal endpoint, ESRD and all-cause mortality [204].

In our longitudinal study performed in the DCCT/EDIC cohort of type 1 diabetes, plasma sphingomyelin was not measured; however, the data are more in line with those of the Steno Diabetes Center [204]. We have found that at baseline, when the patients had normal AER, the patients who later developed diabetic kidney disease (macroalbuminuria) during a 25-year follow-up period had decreased plasma levels of long- and very-long-chain ceramides [189] and lactosylceramides [155], strongly suggesting that low levels of specific ceramides and lactosylceramides could be early markers of kidney damage in type 1 diabetes [155]. We also examined in the same cohort whether plasma hexosylceramides could predict the development of macroalbuminuria and found no association between hexosylceramide levels and development of macroalbuminuria during the 25-year follow-up period [155].

In type 1 diabetes, the decrease in the levels of circulating long- and very-long-chain ceramides and lactosylceramides in patients who do not have a positive biomarker of kidney disease, but progress to macroalbuminuria during follow-up, could be explained by the reduced synthesis of ceramide and glycosphingolipids, by an increased loss in the urine, or by a combination of both. Diabetes induces the downregulation of fatty acid elongases in several tissues, leading to a decreased formation of long- and very-long-chain fatty acids [190,205] and, as a consequence, to a potential decrease in long- and very-long-chain sphingolipids. In diabetes, the downregulation of liver fatty acid elongases may be responsible for the decreased secretion of ceramides and other sphingolipids with very-long fatty acid chains into the circulation. A decrease in very-long-chain sphingolipids is associated in different tissues with a marked increase in vascular permeability [190,206]. Therefore, a decrease in the secretion by the liver of ceramides/sphingolipids with long- and very-long-chain fatty acids may lead to a decrease in the long-/very-long-chain sphingolipids carried by circulating lipoproteins. This decrease may result in increased tissue vascular permeability, including glomerular permeability, and likely to the loss of ceramides/sphingolipids in the urine.

It is possible that the difference in results concerning the levels of sphingomyelin and CKD in type 1 diabetes between the Finish [202,203] and Danish cohorts [204] may result from a difference in the methodology used to measure sphingolipids. The Finish group measured total serum sphingomyelin concentration using proton NMR (nuclear magnetic resonance) metabonomics platform, whereas the Steno group measured concentrations of individual species of each sphingolipid, not total levels of sphingolipids, using non-targeted mass spectrometry serum lipidomic analyses. Our data in type 2 diabetes generated using targeted mass spectrometry plasma lipidomic analyses ([110], see below) clearly show that changes in the levels of the different sphingolipid species carried by the different lipoproteins are more specifically associated with events than total plasma sphingolipid levels; both measures do not necessarily mimic each other.

### 5.3. Type 2 Diabetes Human Studies

Studies on serum/plasma lipidomics and diabetic kidney disease in individuals with type 2 diabetes are limited, with the majority not being focused on sphingolipids and sphingolipid profiling, rather on other classes of lipids in the context of lipogenesis and energy consumption [207,208,209,210].

We have recently conducted a pilot study examining whether plasma levels of sphingolipids (ceramides; sphingoid bases: sphingosine and dihydrosphingosine; and their phosphates: S1P and sphinganine 1-phosphate; sphingomyelin; glycosphingolipids: hexosylceramides; and lactosylceramides) differ between normal healthy controls and participants with diabetes with either normal AER or macroalbuminuria. We also examined whether the differences are mainly associated with changes in the content of sphingolipids carried by the different circulating lipoproteins [110]. We have found that the amounts of all measured ceramide and sphingomyelin species carried by LDL as well as C16:0 hexosylceramide in the diabetic patients with macroalbuminuria were higher compared to controls [110]. The source of the of the LDL-C16:0 hexosylceramide increase in diabetic patients with macroalbuminuria could be attributed to changes in the regulation of ABCA1 transporter in the liver and intestine during the formation of apo B-containing particles, since liver and intestine ABCA1 determines ~80% of plasma hexosylceramides [19]. Notably, the levels of LDL-cholesterol in the two groups of diabetes were lower than in the control group, likely because 75% of the patients were being treated with statins, and only 25% of the control group were on statin therapy. That may indicate that sphingolipid levels do not provide the same message as LDL-cholesterol levels and that statin therapy, although reducing cholesterol levels does not reduce the levels of sphingolipids carried by lipoproteins. Sphingolipids may therefore represent part of the residual CVD risk that statin therapy is not addressing. A recent work by Chapman et al. also raised this possibility [124].

We have also clearly shown that the levels of sphingolipids carried by HDL are also strongly impacted by diabetes [110]. Levels of all ceramide, sphingomyelin and lactosylceramide species carried by both HDL2 and HDL3 were significantly decreased in diabetic subjects with and without macroalbuminuria, when compared to controls. However, the levels of hexosylceramide species carried by both HDL2 and HDL3 in the diabetic patients with normal AER were similar to those of control subjects. When both groups of diabetes were compared to each other, a moderate trend to lower levels in all species of hexosylceramide carried by HDL2 and HDL3 were observed in patients with macroalbuminuria, but only the levels of C16:0, C18:0 and C24:1 hexosylceramides carried by HDL3 were significantly lower [110].

The notion that diabetes reduces the transfer of ceramide, sphingomyelin and lactosylceramide from cells/tissues into HDL is strongly supported by our recent data, which are quite striking [110]. The marked decrease in sphingolipids carried by HDL can be attributed to diabetes-induced changes in the activity of the enzymes involved in the intracellular regulation of these sphingolipids leading to decreased production, decreased efflux, or both. In diabetic patients who developed kidney disease, we found an increase in the levels of C22:0-C26:0 lactosylceramides carried by HDL, but although the levels were higher in the patients with macroalbuminuria than in patients with normal AER, they were still significantly lower than in control subjects [110]. It is possible that kidney damage promotes increased generation and accumulation as well as increased excretion of sphingolipids and glycosphingolipids. In a sub-cohort of the DCCT/EDIC type 1 diabetes cohort, we have found that increased urinary excretion of ceramide, sphingomyelin and lactosylceramide occurs very early in the development of diabetic kidney disease in patients who later developed macroalbuminuria (Lopes-Virella et al., unpublished observations). It has been well documented that an increased accumulation of sphingolipids occurs in diabetic kidney disease [211] and could be a major cause for lipotoxicity [212,213,214].

### 5.4. Conclusions

Our studies suggest that total plasma sphingolipid measurements are not very informative compared to the measurements performed in lipoprotein fractions. Sphingolipidomic analysis of lipoproteins particles, although more laborious, provides a better mechanistic insight of the pathology of diabetic kidney disease than an analysis of total plasma. The relative proportion of sphingolipids carried by each lipoprotein may differentially impact metabolic and signaling pathways at the cell/tissue level, and therefore provide crucial information and a better understanding of the role of sphingolipids in diabetic complications. Supporting this concept, our group has shown that, in podocytes exposed to LDL enriched with specific sphingolipids, the impact on metabolic and signaling pathways is quite distinct from that of HDL enriched with the same sphingolipids [215]. In conclusion, our data in type 2 diabetes and in type 1 diabetes demonstrated that plasma and lipoprotein sphingolipids are associated with diabetic kidney disease. The data also showed that the current knowledge in this field is limited and needs to be expanded by studying the mechanisms involved in the transport of sphingolipids for incorporation into lipoproteins. We believe that our studies opened an important field of investigation that could identify new therapeutic targets.

## 6. Diabetic Retinopathy

Diabetic retinopathy is a complex complication that, in addition to the microvasculature, also affects macroglial cells, microglia cells and neurons. Hyperglycemia and dyslipidemia are the main metabolic abnormalities that affect retinal degeneration in diabetes. Whereas the role of hyperglycemia in inducing diabetic retinopathy has been intensively studied, studies on the effect of dyslipidemia have been scarce. Diabetic dyslipidemia originates from the dysregulation of lipid uptake, metabolism and secretion by adipocytes, as well as by disturbed transport and clearance from circulation. With the development of advanced high-resolution mass spectrometry technology, it is now possible to perform comprehensive lipidomic analyses from limited amounts of postmortem human retina samples or retinas from small animal models. The importance of specific lipid classes (fatty acyls, oxidized bioactive lipids, glycated phospholipids, sterol lipids, and sphingolipids) in diabetic retinopathy was previously highlighted [216]. In fact, our group as part of the DCCT/EDIC Research Group contributed to several seminal publications on the role of modified lipoproteins and lipoprotein-containing immune complexes in retinopathy in type 1 diabetes patients [217,218,219]. We also investigated lipid-mediated mechanisms contributing to diabetic retinopathy in vitro and in animal models [220,221,222,223]. More recently, Lu et al. showed that sphingolipids are involved in the stimulation of cytokine expression by the saturated fatty acid palmitate and lipopolysaccharides (LPS), both implicated in diabetic retinopathy [224]. Using human retinal microvascular endothelial cells, they found that palmitate stimulated ceramide production via both de novo synthesis and hydrolysis of sphingomyelin, and LPS further increased the de novo synthesis of ceramide [224]. They also showed that palmitate induced apoptosis through ceramide production, but the addition of LPS did not enhance apoptotic cell death.

Neovascularization (vasculogenesis and angiogenesis) in ocular tissues can lead to vision-threatening vascular diseases, including the later proliferative stage of diabetic retinopathy. It has been established that S1P acts as an angiogenic factor, promoting embryonic vascular development, and is believed to promote neovascularization through the activation of angiogenic factors such as VEGF (vascular endothelial growth factor), MMP-2 (matrix metalloproteinase-2), and others. The role of S1P, SK2, and opposing roles of S1P receptors in ocular neovascularization has been recently reviewed [225,226]. Although the mechanisms underlying the S1P regulation in pathological neovascularization remain elusive, S1P signaling remains as a promising therapeutic target to suppress pathological retinal neovascularization.

Elongases are highly expressed in the retina and they are actively involved in the de novo synthesis of saturated, monounsaturated, and polyunsaturated fatty acids. Very long-chain fatty acids that incorporate into very long-chain ceramides are produced by the elongase ELOVL4 (elongation of very long-chain fatty acids protein 4). ELOVL4 is the highest expressed elongase in the retina and elongates very long-chain fatty acids ≥C24 to produce ≥C26 very long-chain polyunsaturated and saturated fatty acid. ELOVL4 is significantly reduced in the retina of diabetes patients. Kady et al. showed that overexpression of ELOVL4 in bovine retinal endothelial cells significantly decreased basal permeability, inhibited VEGF- and IL-1β-induced permeability, and prevented VEGF-induced decrease in tight junction proteins [190]. Intravitreal delivery of AAV2-hELOVL4 reduced the diabetes-induced increase in vascular permeability [190]. Thus, through the increase in very-long-chain ceramides and the stabilization of the tight junction, normalization of retinal ELOVL4 expression could restore the integrity of the blood–retinal barrier in diabetic retinopathy.

Serine is a non-essential amino acid directly involved in cellular homeostasis, proliferation, and differentiation, and other than being an integral amino acid in multiple essential proteins, free L-serine is essential for generating sphingolipids (Figure 1). Studies have demonstrated a correlation between serine deficiency and systemic diabetes (reviewed in [227]). Exogenous supplementation of serine has proven to be effective in many cases, reducing oxidative stress and reducing cytokine levels, but endogenous synthesis of serine augments inflammatory responses [228]. Importantly, multiple studies have found that if diabetic patients are treated with a serine supplement, blood glucose levels were reduced [228,229].

Although the mechanisms involved in the reduction of glucose mediated by administration of serine is not known, recent studies may have uncovered a possible explanation that needs to be further investigated. These studies found that the alanine/serine/cysteine transporter 1 (ASC-1), which is highly expressed in adipocytes, is an important modulator of thermogenesis [230], lipid metabolism, lipid storage and insulin resistance [231,232]. Although initially thought to be only present in white pre-adipocytes, it is now considered to be an important and complex metabolic regulator in different adipose tissue depots and adipocyte subtypes. Overexpression of the ASC-1 transporter was found to lower the generation of ROS and stimulate the respiratory mitochondrial activity, and its deficit or inhibition exhibits the opposite effect. Therefore, it is possible that administration of serine may somewhat lead to an increase in serine transport and slight improvement in insulin resistance.

The retina relies most heavily on serine, and any deficiencies may cause the tissue to exhibit the first signs of disease in diabetic retinopathy [233]. It is also known that upon onset of diabetes, toxic sphingolipid (deoxysphingolipids) starts accumulating causing symptoms similar to serine deficiency [234] (see the section on neuropathy).

## 7. Diabetic Neuropathy

Glycosphingolipids are a diverse group of cell membrane components, and their metabolites have a role in intercellular communication, functioning as biochemical signals involved in many cellular pathways. Gangliosides and sulfatides are key constituents of the neuronal plasma membrane and myelin, respectively. Little is known about the complex regulation of the individual metabolic steps of ganglioside and glycosphingolipids; however, the main biosynthetic and catabolic pathways seem to be regulated at genetic and/or posttranslational levels (reviewed in [235]). Factors like membrane fluidity, lipid and protein composition of organellar membranes, and nutritional state have been recognized as important regulators of the biosynthetic metabolic pathways of glycosphingolipids as well as gangliosides. For example, a high-fat (Western) diet may cause increased production of C16-sphingolipids [236], including incorporation into ganglioside GM3, which in turn can downregulate insulin receptor activity, thereby exacerbating metabolic syndrome and type 2 diabetes mellitus [237]. In the 1980s, experimental animal studies showed that ganglioside treatment corrects deficits of nerve conduction velocity and prevents impairments in regeneration from a sciatic nerve crush (reviewed in [238]). Unfortunately, clinical trials in humans showed the uselessness of ganglioside administration (reviewed in [239,240]). In fact, the mixed bovine gangliosides have been withdrawn from the market in Europe because of side effects (e.g., Guillain–Barre syndrome) [240].

Serine palmitoyl transferase (SPT) is a rate-limiting enzyme which regulates de novo sphingolipid biosynthesis (Figure 1). Dysfunctional genes in SPT can lead to the neurodegeneration hereditary sensory and autonomic neuropathy type 1 (HSAN1) disease [241]. Mutations either in subunit SPTLC1 or SPTLC2 cause a higher incorporation rate for other amino acids than L-serine, mainly L-alanine and glycine, and cause increased levels of 1-deoxysphingolipids, mainly 1-deoxydihydroceramides, which cannot be converted to sphingomyelin, glycosphingolipids, or gangliosides. Deoxysphingolipids were shown to aggregate and induce apoptosis in in vitro and in vivo models [242] For example, retinal organoids treated with deoxysphingolipids exhibited dose-dependent apoptosis [227], and when located to the mitochondria they caused mitochondrial dysfunction [243]. Our group has recently shown that deoxysphingolipids upregulated MMP-1, downregulated TIMP-1, and induced cytotoxicity in Schwann cells in vitro [244]. Furthermore, sphingolipidomic analysis of lipoproteins from type 2 diabetes patients with and without neuropathy showed that only HDL2 isolated from those patients with neuropathy contained a higher level of deoxysphingolipids compared to patients without neuropathy. Notably, HDL2 isolated from type 2 diabetes patients with neuropathy was more potent compared to HDL2 from patients without neuropathy in upregulating MMP-1, downregulating TIMP-1, and stimulating collagenase activity in Schwann cells [244]. Our data demonstrated a potential causal relationship of deoxysphingolipids in diabetic neuropathy.

As mentioned above (the Retinopathy section), multiple studies have found that if diabetic patients are treated with a serine supplement, blood glucose levels were reduced [228,229]. Analysis of deoxysphingolipids in plasma revealed that they are present at low levels in plasma from normal healthy individuals, primarily in VLDL and LDL [241]. However, plasma levels of deoxysphingolipids are elevated in type 2 diabetes patients [90,130,234] and in patients exhibiting symptoms of the metabolic syndrome [130]. Furthermore, Wei et al. reported that plasma deoxy-sphingoid bases are elevated in type 2 diabetes patients and correlate with the stage of diabetic distal sensorimotor polyneuropathy [90]. The same authors also reported that levels of deoxy-sphingoid bases in type 1 diabetes patients do not differ from those in control subjects, but they did not measure deoxysphingolipids levels in type 1 diabetes patients with neuropathy.

Our group investigated the associations between plasma levels of multiple sphingolipid species, including deoxysphingolipids, and free amino acids, and the presence of symptomatic neuropathy in a DCCT/EDIC type 1 diabetes sub-cohort (n = 80) [245]. Patient-determined neuropathy was based on a 15-item self-administered questionnaire (Michigan Neuropathy Screening Instrument) developed to assess distal symmetrical peripheral neuropathy in diabetes [245]. Patients who scored ≥4, or reported an inability to sense their feet during walking or to distinguish hot from cold water while bathing were considered neuropathic. Plasma levels of ceramide, sphingomyelin, hexosylceramide and lactosylceramide species, and amino acids were measured and analyzed relative to neuropathy status of the patient. We found that levels of deoxy-C24:0-ceramide, C24:0 and C26:0 ceramide were higher in patients with neuropathy than those without neuropathy and levels of cysteine, but not any other measured amino acid, were higher in patients with neuropathy. Therefore, plasma deoxy-ceramide and ceramide species may have potential diagnostic and prognostic significance in diabetic neuropathy.

## 8. Conclusions

This review summarizes the present knowledge concerning the mechanisms by which sphingolipids contribute to the development of insulin resistance, type 1 and type 2 diabetes and their complications. Sphingolipids have emerged in the last decade as new risk factors in the development of type 1 and type 2 diabetes and their complications. Furthermore, sphingolipidomics are filling an important gap in the cardiometabolic field of research due to the crucial role of sphingolipids in inflammation and metabolic pathways.

Initially, most of the published work was focused on tissue sphingolipids, not sufficiently on circulating sphingolipids. In this review, we have expanded the scope of the past literature by focusing not only on circulating sphingolipids, but on the lipoproteins that carry them, and on the differential disease impact of the different species of sphingolipids. Despite the progress, the field is still in its infancy and further studies are needed to examine in greater detail the abnormalities of circulating sphingolipids, the mechanisms involved, and sphingolipid associations with the different stages of the metabolic syndrome, type 1 and type 2 diabetes, and their complications.

Our studies in type 1 diabetes, using the DCCT/EDIC cohort, showed that most of the sphingolipid changes occur very early in the development of CKD, strongly suggesting that with early intervention, we may be able to prevent or reduce the development of CKD in type 1 diabetes. If further studies in type 2 diabetes and in other diabetic complications show such an early trend of detection, the use of sphingolipids as an early biomarker of disease will be extremely promising. More than serving as biomarkers, alterations in sphingolipids may uncover targeted therapeutic interventions that can prevent the development of diabetes and its complications.

## Data Availability

Not applicable.

## Abbreviations

ABCA1	ATP-binding cassette family A protein1
ABCC10	ATP-binding cassette family C protein10
AER	Albumin excretion rate
Akt/PKB	Serine/threonine kinase, also known as protein kinase B
Apo B	Apolipoprotein B
Apo M	Apolipoprotein M
BMI	Body mass index
C1P	Ceramide 1-phosphate
CerS	Ceramide synthase
CVD	Cardiovascular disease
DAG	Diacylglycerol
DCCT/EDIC	Diabetes Control and Complications Trial/Epidemiology of Diabetes Interventions and Complications
eGFR	Estimated glomerular filtration rate
ELOVL	Elongation of very long-chain fatty acids protein
ER	Endoplasmic reticulum
ESRD	End-stage renal disease
FFA	Free fatty acids
HbA1c	Hemoglobin A1C
HDL	High-density lipoproteins
HPLC-MS/MS	High performance liquid chromatography-Tandem mass spectrometry
IDL	Intermediate-density lipoproteins
IFN	Interferon
IL-1	Interleukin-1
IRS-1	Insulin receptor substrate 1
LC3B	Acetylated microtubule-associated protein 1 light chain 3B
LDL	Low-density lipoproteins
Mfsd2b	Major facilitator superfamily domain-containing protein 2B
MMP	Matrix metalloproteinase
MTP	Microsomal triglyceride transfer protein
NAFL	Non-alcoholic fatty liver
NASH	Non-alcoholic steatohepatitis
NOD	Non-obese diabetic
ORMDL3	Orosomucoid like 3; ORMDL sphingolipid biosynthesis regulator 3
PI3K	Phosphatidylinositol 3-kinase
PIP2	Phosphatidylinositol 4,5 bisphosphate
PIP3	Phosphatidylinositol (3,4,5)-triphosphate
PKC	Protein kinase C
PP2A	Protein phosphatase A2
ROS	Reactive oxygen species
S1P	Sphingosine 1-phosphate
SK	Sphingosine kinase
SLE	Systemic lupus erythematosus
SPL	Sphingosine phosphate lyase
SPP2	Secreted phosphoprotein 2
SPNS2	Spinster homologue 2
SPT	Serine palmitoyl transferase
STZ	Streptozotocin
TIMP	Tissue inhibitor of metalloproteinase
TLR4	Toll-like receptor 4
TNF	Tumor necrosis factor
VEGF	vascular endothelial growth factor
VLDL	Very-low-density lipoproteins
